# Lanthanoid-free perovskite oxide catalyst for dehydrogenation of ethylbenzene working with redox mechanism

**DOI:** 10.3389/fchem.2013.00021

**Published:** 2013-10-23

**Authors:** Ryo Watanabe, Maiko Ikushima, Kei Mukawa, Fumitaka Sumomozawa, Shuhei Ogo, Yasushi Sekine

**Affiliations:** ^1^Department of Materials Science and Chemical Engineering, Faculty of Engineering, Shizuoka UniversityHamamatsu, Japan; ^2^Department of Applied Chemistry, School of Science and Engineering, Waseda UniversityTokyo, Japan

**Keywords:** dehydrogenation of ethylbenzene, perovskite oxide catalyst, redox mechanism, stable under severe conditions, styrene production, lattice oxygen

## Abstract

For the development of highly active and robust catalysts for dehydrogenation of ethylbenzene (EBDH) to produce styrene; an important monomer for polystyrene production, perovskite-type oxides were applied to the reaction. Controlling the mobility of lattice oxygen by changing the structure of Ba_1 − *x*_Sr_x_Fe_*y*_Mn_1 − *y*_O_3 − δ_ (0 ≤ *x* ≤ 1, 0.2 ≤ *y* ≤ 0.8), perovskite catalyst showed higher activity and stability on EBDH. The optimized Ba/Sr and Fe/Mn molar ratios were 0.4/0.6 and 0.6/0.4, respectively. Comparison of the dehydrogenation activity of Ba_0.4_Sr_0.6_Fe_0.6_Mn_0.4_O_3 − δ_ catalyst with that of an industrial potassium promoted iron (Fe–K) catalyst revealed that the Ba_0.4_Sr_0.6_Fe_0.6_Mn_0.4_O_3 − δ_ catalyst showed higher initial activity than the industrial Fe–K oxide catalyst. Additionally, the Ba_0.4_Sr_0.6_Fe_0.6_Mn_0.4_O_3 − δ_ catalyst showed high activity and stability under severe conditions, even at temperatures as low as 783 K, or at the low steam/EB ratio of 2, while, the Fe–K catalyst showed low activity in such conditions. Comparing reduction profiles of the Ba_0.4_Sr_0.6_Fe_0.6_Mn_0.4_O_3 − δ_ and the Fe–K catalysts in a H_2_O/H_2_ atmosphere, reduction was suppressed by the presence of H_2_O over the Ba_0.4_Sr_0.6_Fe_0.6_Mn_0.4_O_3 − δ_ catalyst while the Fe–K catalyst was reduced. In other words, Ba_0.4_Sr_0.6_Fe_0.6_Mn_0.4_O_3 − δ_ catalyst had higher potential for activating the steam than the Fe–K catalyst. The lattice oxygen in perovskite-structure was consumed by H_2_, subsequently the consumed lattice oxygen was regenerated by H_2_O. So the catalytic performance of Ba_0.4_Sr_0.6_Fe_0.6_Mn_0.4_O_3 − δ_ was superior to that of Fe–K catalyst thanks to the high redox property of the Ba_0.4_Sr_0.6_Fe_0.6_Mn_0.4_O_3 − δ_ perovskite oxide.

## Introduction

Styrene, an important monomer in petrochemistry, is used for polymeric materials such as polystyrene resin, acrylonitrile–butadiene–styrene resin and styrene–butadiene rubber. The production volume of styrene is 30 million tons per year worldwide (Meima and Menon, 2001; Su et al., 2005; Won and Jang, 2011). Styrene is produced via catalytic dehydrogenation of ethylbenzene (EBDH) according to the following chemical equation (Equation 1) (Cavani and Trifirò, 1995).

(1) $${\text{C}}_{8}{\text{H}}_{10}\to {\text{C}}_{8}{\text{H}}_{8}+{\text{H}}_{2}$$

As an endothermic reaction, EBDH requires high temperatures for high conversion of ethylbenzene because of thermodynamic limitations. An iron-based catalyst promoted by potassium and many kinds: Cr_2_O_3_, MoO_3_, CeO_2_, and Pd is used as an industrial catalyst (Kearby, 1945; Eggertsen and Voge, 1947; Pitzer, 1958; Lee, 1974; O'Hara, 1975; Riesser, 1979; Hirano, 1986; Rokicki et al., 2004). In the industrial process, steam is supplied with EB for increasing the equilibrium conversion by decreasing the partial pressure of EB. Additionally, steam has roles of heating up the reactant fluid, supplying heat for the endothermic reaction, and inhibiting coke deposition on the catalyst. A disadvantage of EBDH with steam is a large amount of energy loss because of the supply of superheated steam. Therefore, development of a catalyst that can work under low steam conditions and at low temperatures has been pursued.

For energy resource conservation, oxidative dehydrogenation of ethylbenzene (ODH) has recently been emphasized and investigated widely. Because ODH is an exothermic reaction, high conversion can be achieved at lower temperatures than from non-oxidative dehydrogenation. Meso-structured CeO_2_ (Xu et al., 2009), V_2_O_5_/CeO_2_/Al_2_O_3_ (Reddy et al., 2007), and Mg(VO_4_)_2_-MgO (Chang et al., 1995) catalysts were reported as highly active catalysts working at low temperatures of around 723 K for ODH. Onion-like carbon (Su et al., 2005, 2007, 2010) and carbon fibers (Zhao et al., 2007) have also been reported as catalysts showing high activity for ODH. However, because of the combustion of EB and styrene, ODH processes presented some problems such as the decrease of selectivity to styrene. Therefore, the selectivity to styrene was low in the ODH process: about 68% at the EB conversion rate of 91% (Keller et al., 2002). An application of N_2_O and CO_2_ to ethylbenzene dehydrogenation has been conducted to attain high selectivity to styrene, N_2_O, and CO_2_ were used instead of O_2_ to avoid the combustion of styrene and EB, to CO and CO_2_ (Sugino et al., 1995; Sakurai et al., 2000a, 2002; Shiju et al., 2005). As for using N_2_O as the oxidant for ODH of ethylbenzene, high styrene yield was obtained at low temperature, however the selectivity to styrene was low due to the production of styrene oxide as well as benzene and toluene (Shiju et al., 2005). Vislovskiy et al. (2002) and Park et al. (2003) investigated EBDH in the presence of CO_2_ over V–Sb/Al-oxide catalyst. They stated that a redox-type mechanism proceeded on V–Sb/Al-oxide catalyst, which achieved high activity and selectivity to styrene. CO_2_ was considered to be a desirable oxidant for EBDH. Although high activity and stability was acquired over activated carbon-supported vanadium catalyst which was promising dehydrogenation catalyst, deactivation proceeded on the catalyst due to coke deposition. Sakurai et al. investigated the catalytic properties of V/AC catalyst for EBDH with CO_2_ (Sakurai et al., 2000b). The catalyst revealed high activity and selectivity to styrene, but deactivation was not prevented. From these backgrounds, development of a novel dehydrogenation catalyst which has high stability as well as high activity is considered to be required.

We previously investigated the reaction mechanism of EBDH with steam over the industrial potassium promoted the iron catalyst (Fe–K) catalyst, and found for the first time that oxidative dehydrogenation of EBDH (Equation 2) proceeded on the Fe–K catalyst and H_2_O can regenerate the consumed lattice oxygen in the catalyst (Equation 3) (Sekine et al., 2008).

(2) $${\text{C}}_{8}{\text{H}}_{10}+{\text{O}}_{\text{lat}}^{2-}\to {\text{C}}_{8}{\text{H}}_{8}+{\text{H}}_{2}\text{O}+V_{ox}+2{\text{e}}^{-}$$

(3) $${\text{ H}}_{2}\text{O}+V_{ox}+2{\text{e}}^{-}\to {\text{H}}_{2}+{\text{O}}_{\text{lat}}^{2-}$$

Here, O^2−^_lat_ denotes the lattice oxygen and *V*_*ox*_ shows the lattice vacancy in the catalyst. In other words, the catalytic activity and stability depended on redox characteristics of the lattice oxygen in the catalyst. Therefore, investigations of catalytic activity for EBDH with steam over some perovskite-type oxides whose mobility of lattice oxygen in perovskite-type oxide could be controlled, were conducted. As a result of investigations, La_0.8_Ba_0.2_Fe_0.4_Mn_0.6_O_3 − δ_ (LBFMO) catalyst had high activity and stability and revealed superior performance to the Fe–K catalyst at 813 K (Watanabe et al., 2009). In addition, we found that oxidative dehydrogenation using lattice oxygen (Equation 2) proceeded and H_2_O could regenerate the consumed lattice oxygen in the catalyst (Equation 3). The high regeneration rate of lattice oxygen enhanced the activity and stability of EBDH (Watanabe et al., 2011).

However, elemental La contained in LBFMO catalyst is an expensive rare-earth metal that is distributed unevenly throughout the world. Therefore, lanthanoid elements such as La are best not included in the catalyst for industrial applications. Additionally, the catalyst must work under low reaction temperatures and low steam conditions from the viewpoint of energy saving. These severe operations might be possible thanks to high redox property of perovskite-type oxides, which could achieve a low-cost dehydrogenation process.

In this work, to develop a novel La-free perovskite-type oxide catalyst, BaMnO_3 − δ_-based catalysts that are well known for high redox properties, were applied to EBDH with steam. For acquiring high-activity catalysts, the low-valence metal ion such as Ba^2+^, Sr^2+^, or Ca^2+^ was incorporated in A-site in the structure. The valence of B-site cation was fixed at higher-valence state for keeping the charge neutralization conditions by introduction of the low-valence metal ion in A-site, which affected enhancement of the catalytic property of the catalyst. In addition, introducing the low-valence metal ion into the A-site in perovskite was expected to produce oxygen vacancies in the structure, and it would bring high oxygen ionic conductivity. We optimized the structure of the BaMnO_3 − δ_-based catalyst by the substitution of A-site with Sr and B-site with Fe. Additionally, the activity and robustness of the optimized catalyst at low reaction temperature and low steam/EB condition were examined for exploring the possibility as the industrial catalyst.

## Experimental

### Catalyst preparation

Screening tests revealed that Ba-Ca-Fe-Mn-Ox and Ba-Sr-Fe-Mn-Ox perovskite were active and selective catalysts for EBDH as shown in Table 1. Therefore, we used some perovskite oxides in this study: Ba_0.2_Ca_0.8_Fe_0.4_Mn_0.6_O_3 − δ_ and Ba_1 − *x*_Sr_*x*_Fe_*y*_Mn_1 − *y*_O_3 − δ_ (*x* = 0, 0.2, 0.4, 0.6, 0.8, and 1, *y* = 0, 0.2, 0.4, 0.6, and 0.8). They were prepared using a complex polymerization method as follows: Ba(NO_3_)_2_, Sr(NO_3_)_2_, (or Ca(NO_3_)_2_ · 4H_2_O), Fe(NO_3_)_2_ · 9H_2_O, and Mn(NO_3_)_2_ · 6H_2_O (Kanto Kagaku) were dissolved in distilled water. Then, citric acid and ethylene glycol were added to the solution to produce a molar ratio of total metal ions: citric acid: ethylene glycol = 1: 3: 3. The obtained solution was dried up to produce a gel at 353 K. Then the gel was pre-calcined at 673 K for 2 h, and then calcined at 1123 K for 10 h.

**Table 1:** **EB conversion and selectivity to products for EBDH over perovskite catalysts**.

**Catalyst**	**EB Conv./% (0.5 h)**	**Selectivity (0.5 h)/%**		
		**Styrene**	**Benzene**	**Toluene**
CaFe_0.4_Mn_0.6_O_3 − δ_	4.9	77.9	17.3	4.8
SrFe_0.4_Mn_0.6_O_3 − δ_	17.1	91.9	6.5	1.7
BaFe_0.4_Mn_0.6_O_3 − δ_	33.2	94.4	2.4	3.2
Ba_0.2_Ca_0.8_Fe_0.4_Mn_0.6_O_3 − δ_	28.2	96.6	2.0	1.4
Ba_0.2_Sr_0.8_Fe_0.4_Mn_0.6_O_3 − δ_	28.8	95.4	2.9	1.7

### Characterization of the catalyst

The crystalline structure of the prepared catalyst was ascertained using X-ray powder diffraction with CuKα radiation (λ = 1.54 Å, Rint-2000; Rigaku Corp.). The specific surface area of perovskite oxide was measured using N_2_ adsorption at 77 K using Autosorb-1 (Quantachrome Corp.). The sample was outgassed at 573 K for 2 h before N_2_ adsorption. Redox property of the catalyst was measured using thermogravimetric analysis with TGA-50 (Shimadzu Corp.). The catalyst sample was set on the center of the muffle. Then the temperature of the catalyst was raised with a 10 K min^−1^ heating rate from room temperature to 783 K in N_2_ atmosphere. Thermogravimetric measurements were conducted under 10 vol% H_2_ in N_2_ atmosphere and also conducted under 10 vol% H_2_ + 1 vol% H_2_O or 2.5 vol% H_2_O in N_2_ atmosphere at 783 K to elucidate the effect of steam on the reduction profile of the catalyst. Weight loss “0” was the starting weight of the catalyst.

### Activity test

Catalytic activity, selectivities to products and stability were examined in a conventional fixed bed reactor. The reactor used for this study comprised a quartz tube (10-mm o.d.) containing a catalyst bed, which was fixed by quartz wool. A type-K thermocouple enclosed in a quartz thermowell (3-mm o.d.) was positioned inside the catalyst bed for the measurement of the catalyst temperature. Reactions were conducted at 783 or 813 K at atmospheric pressure in the presence of steam; the molar ratio of steam to ethylbenzene was 2 or 12. The weight hourly space velocity (WHSV) was 1.2 h^−1^; the catalyst weight was 1 g. Liquid products such as ethylbenzene, benzene, toluene, and styrene were analyzed using off-line flame ionization detection (FID) gas chromatograph (GC-2014; Shimadzu Corp.). Gaseous products such as H_2_, CO, and CH_4_ were analyzed using off-line thermal conductivity detection (TCD) gas chromatograph (GC-8A; Shimadzu Corp.). The conversion (Equation 4) and styrene yield (Equation 5) were evaluated using following equations.

(4) $$\begin{array}{l} \text{EB conversion}[/\%]=([\text{Sty}]+[\text{Bz}]+[\text{Tol}])/{\left([\text{EB}]+[\text{Sty}]+[\text{Bz}]+[\text{Tol}]\right)}^{*}100 \end{array}$$

(5) $$\begin{array}{l} \text{Styrene yield}[/\%]=\;[\text{Sty}]/{\left([\text{Sty}]+[\text{Bz}]+[\text{Tol}]\right)}^{*}(\text{EBconversion}) \end{array}$$

(6) $$\text{Styrene selectivity}[/\%]=\;[\text{Sty}]/{\left([\text{Sty}]+[\text{Bz}]+[\text{Tol}]\right)}^{*}100$$

Here, [EB], [Sty], [Bz], and [*Tol*] were, respectively, the concentration of EB, styrene, benzene and toluene in the effluent gas. Carbon balances in this work were over 95% through all the experimentally obtained results.

### XPS measurement for characterization of catalyst surface

X-ray photoelectron spectroscopy (XPS, ESCA1800; Ulvac-Phi Inc.) measurement was performed using a non-monochromatic Al-Kα radiation. The pass energy of the analyzer was set at 23.5 eV. Binding energies obtained for an identical sample were reproducible within ±0.1 eV in general. Binding energy of C1s was corrected at 284.7 eV.

### Surface exchange reaction

For clarifying redox properties of perovskite catalyst, surface exchange reaction using isotope was performed as the following method. First, 100% of H^16^_2_O was supplied to the catalyst for 30 min in order to fill ^16^O^2−^ in the perovskite. After adequately feeding H^16^_2_O in the catalyst, the mixed gas of H^18^_2_O (30%) and H^16^_2_O (70%) was fed to the catalyst as a step at 783 K for substitution of lattice oxygen from ^16^O^2−^ to ^18^O^2−^. Temporal changes of concentrations of H^18^_2_O and H^16^_2_O were monitored using a quadruple mass spectrometer (Q-Mass).

## Results and discussion

### Dehydrogenation activity and selectivity to products over BaMnO_3 − δ_-based catalysts

Previous studies revealed that Mn-based and Fe/Mn-oxide catalysts showed high activity for EBDH thanks to the well-balanced rates of release and regeneration of lattice oxygen (Watanabe et al., 2009, 2011). In addition, Ba-based perovskite was well known to give better performance for oxygen-ion diffusivity (Wang et al., 2005; Vente et al., 2006; Zeng et al., 2007; Cheng et al., 2008). As mentioned in the introduction, high oxygen ionic conductivity was obtained by incorporating low-valence metal ions, such as Ba^2+^, Sr^2+^, and Ca^2+^into the A-site in perovskite-type oxides. As shown in Table 1, BaMnO_3 − δ_-based catalysts showed high activity, compared with CaMnO_3 − δ_-based and SrMnO_3 − δ_-based catalyst. Incorporation of the alkaline earth metal of Ba in perovskite structure would lead to the generation of electron holes and oxygen vacancies as the charge compensation, which could induce the high oxygen mobility derived from the mixed conduction by electrons and oxygen ions. Also, the incorporation of Ba in perovskite structure would engender the large free volume in the lattice, which could decrease the activation energy of oxygen ion migration. Therefore, to develop novel La-free perovskite catalysts for EBDH, BaMnO_3 − δ_-based perovskite oxides were applied as catalysts.

Table 2 presents results for catalytic activities over BaMnO_3 − δ_, Ba_0.2_Sr_0.8_MnO_3 − δ_, and Ba_0.2_A_0.8_Fe_0.4_Mn_0.6_O_3 − δ_(A = Ca^2+^, Sr^2+^) at 813 K with the steam/EB molar ratio of 12. Styrene yield over BaMnO_3 − δ_, Ba_0.2_Sr_0.8_MnO_3 − δ_, Ba_0.2_Ca_0.8_Fe_0.4_Mn_0.6_O_3 − δ_, and Ba_0.2_Sr_0.8_Fe_0.4_Mn_0.6_O_3 − δ_ catalysts at 0.5 h were, respectively 22.0, 27.1, 26.9, and 27.5%. BaMnO_3 − δ_-based catalysts showed high activity for EBDH. The substitution of Sr^2+^ for Ba^2+^enhanced dehydrogenation activity; however, the stability was not improved by the substitution. The reason for improving high catalytic activity by the substitution of Sr^2+^ for Ba^2+^ might be enhancement of high releasing rate of lattice oxygen (Equation 2). In other words, ionic conductivity might increase by the substitution of Sr^2+^. Shao et al. investigated the O_2_ permeability property of Ba_*x*_Sr_1 − *x*_Co_0.8_Fe_0.2_O_3 − δ_ (*x* = 0−1) perovskite membrane (Shao et al., 2001). They concluded that there existed an optimal value of Ba^2+^ substitution ratio (*x* = 0.3) in terms of O_2_ permeability because of the low activation energy of oxygen transportation in Ba_0.3_Sr_0.7_Co_0.8_Fe_0.2_O_3 − δ_ perovskite membrane. The slightly substitution of Sr^2+^ for Ba^2+^ might decrease an activation energy for releasing rate of lattice oxygen. Therefore, initial dehydrogenation activity of the Ba_0.2_Sr_0.8_MnO_3 − δ_ catalyst was higher that of the BaMnO_3 − δ_ catalyst. However, the rate for regenerating lattice oxygen (Equation 3) might not be improved, so stability was not improved.

**Table 2:** **Durability and the amount of deposited carbon on Ba_1 − *x*_Sr_*x*_Fe_0.4_Mn_0.6_O_3 − δ_ (0 ≤ *x* ≤ 1) catalyst**.

**Catalyst**	Durability^*^	**Amount of carbon deposition/mg g-cat^−1^**
BaFe_0.4_Mn_0.6_O_3 − δ_	0.48	6.4
Ba_0.8_Sr_0.2_Fe_0.4_Mn_0.6_O_3 − δ_	0.37	7.4
Ba_0.6_Sr_0.4_Fe_0.4_Mn_0.6_O_3 − δ_	0.46	4.6
Ba_0.4_Sr_0.6_Fe_0.4_Mn_0.6_O_3 − δ_	0.59	4.8
Ba_0.2_Sr_0.8_Fe_0.4_Mn_0.6_O_3 − δ_	0.65	5.8
SrFe_0.4_Mn_0.6_O_3 − δ_	0.60	7.6

* Durability = Styrene yield at 2 h/Styrene yield at 0.5 h.

While, the substitution of Fe for Mn improved the stability, and the initial activity showed similar values on Ba_0.2_Sr_0.8_MnO_3 − δ_, and Ba_0.2_Sr_0.8_Fe_0.4_Mn_0.6_O_3 − δ_ catalysts. Comparison of the catalytic performance between Ba_0.2_Ca_0.8_Fe_0.4_Mn_0.6_O_3 − δ_ and Ba_0.2_Sr_0.8_Fe_0.4_Mn_0.6_O_3 − δ_, a catalyst substituted with Sr (Ba_0.2_Sr_0.8_Fe_0.4_Mn_0.6_O_3 − δ_) showed better activity and stability. Ba_0.2_Sr_0.8_Fe_0.4_Mn_0.6_O_3 − δ_ catalyst showed comparable initial activity with Fe–K catalyst. The incorporation of Fe in perovskite-type oxide was found to suppress the lattice oxygen release (Equation 2) and promote the lattice oxygen regeneration (Equation 3) in the previous investigation (Watanabe et al., 2011). Therefore, the Fe/Mn-oxide catalyst showed higher stability for EBDH than Mn-based oxide thanks to the well-balanced rates of release and regeneration of lattice oxygen.

Styrene selectivity over Ba_0.2_Sr_0.8_MnO_3 − δ_ catalyst was 94.1 % and that over Ba_0.2_Sr_0.8_Fe_0.4_Mn_0.6_O_3 − δ_ was 95.4%. Introducing Fe in the B-site of Ba_0.2_Sr_0.8_MnO_3 − δ_ perovskite catalyst enhanced the activity, stability, and selectivity to styrene. After 2 h of reaction, styrene selectivity was improved a little. The reason for improvement of the styrene selectivity of all catalysts in Table 3 after 2 h of reaction was that the formation rate of benzene and toluene degraded faster than that of styrene formation rate. Further investigations by optimizing the structure of Ba-Sr-Fe-Mn-perovskite catalyst were conducted to improve the activity/selectivity to styrene.

**Table 3:** **Catalytic performances for EBDH over perovskite catalysts: reaction times**.

**Catalyst**	**BET specific surface area/m^2^ g^−1^**	**Styrene yield/%**		**Styrene selectivity/%**	
		**at 0.5 h**	**at 2.0 h**	**at 0.5 h**	**at 2.0 h**
BaMnO_3 − δ_	2.8	22.0	5.1	94.5	96.0
Ba_0.2_Sr_0.8_ MnO_3 − δ_	8.4	27.1	3.6	94.1	94.0
Ba_0.2_Ca_0.8_ Fe_0.4_Mn_0.6_O_3 − δ_	9.7	26.9	13.2	96.6	97.8
Ba_0.2_Sr_0.8_ Fe_0.4_Mn_0.6_O_3 − δ_	13.1	27.5	17.9	95.4	97.8
Fe–K	2.0	27.1	29.4	97.8	98.3

### Optimization of A-site substitution ratio of Sr^2+^ for Ba^2+^ in Ba_1 − x_Sr_x_Fe_0.4_Mn_0.6_O_3 − δ_ catalyst for EBDH

**Figure 1:**
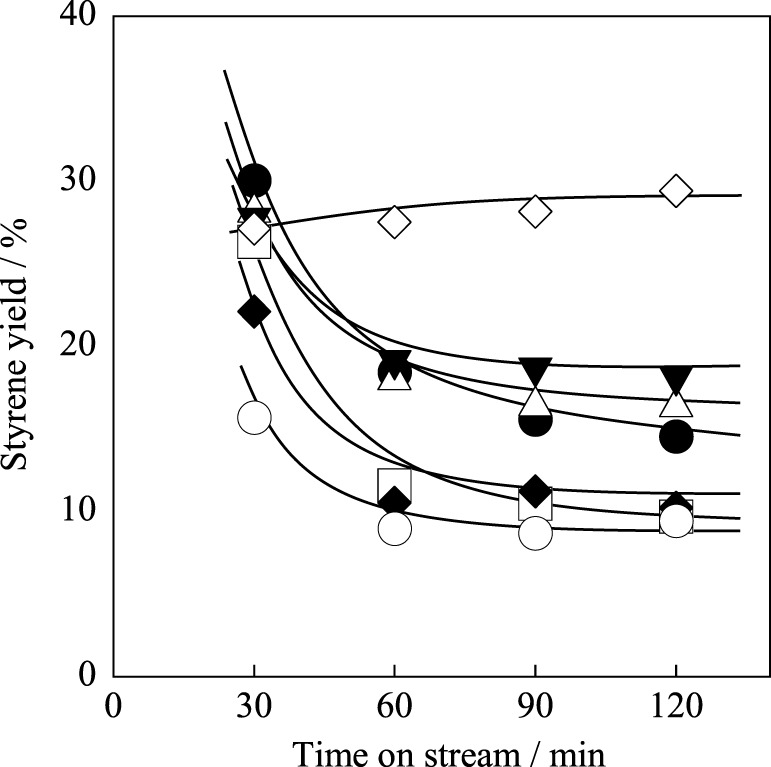
**Effect of Sr^2+^ substitution ratio in Ba_1 − *x*_Sr_*x*_Fe_0.4_Mn_0.6_O_3 − δ_ catalyst on styrene yield: (•) *x* = 0, (□) *x* = 0.2, (♦) *x* = 0.4, (∆) *x* = 0.6, (▼) *x* = 0.8, (○) *x* = 1.0, and (◊) Fe–K catalyst**.

**Figure 2:**
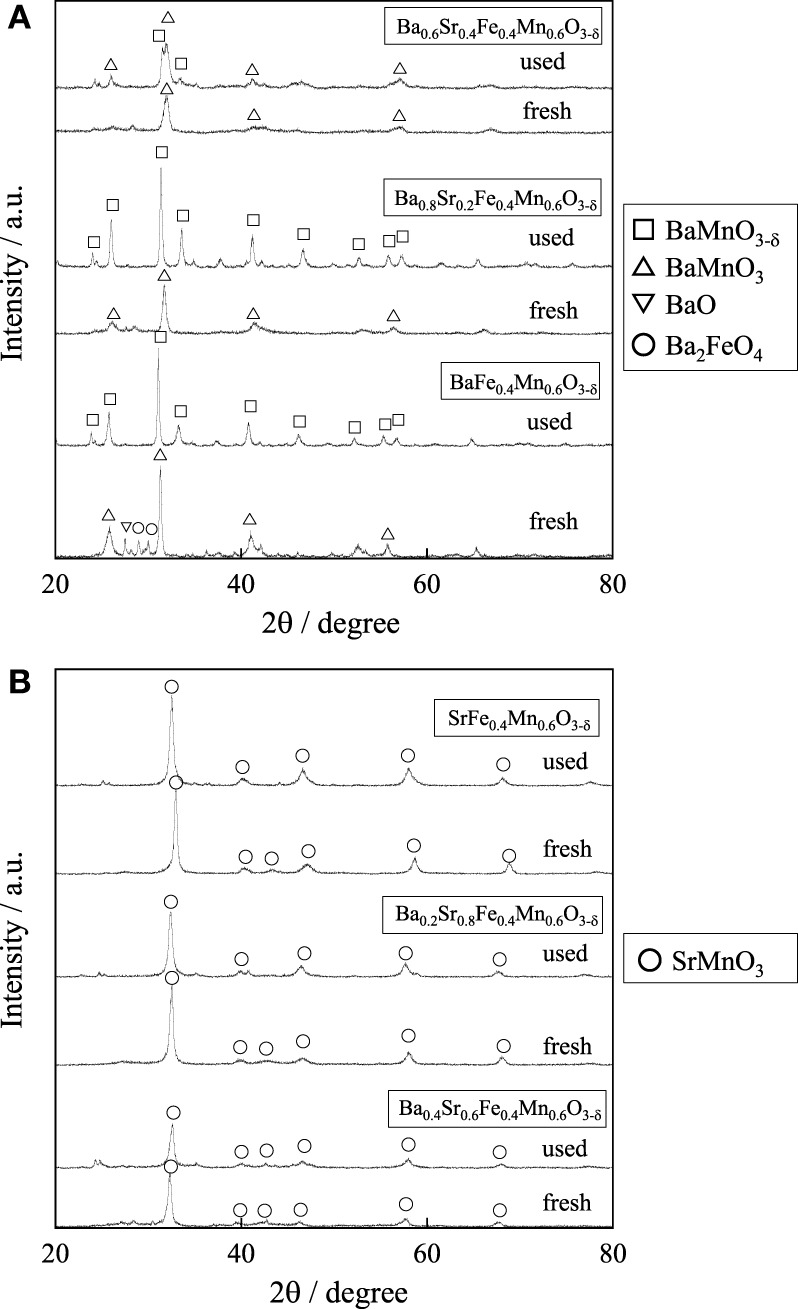
**XRD patterns for (A) Ba_1 − *x*_Sr_*x*_Fe_0.4_Mn_0.6_O_3 − δ_ (0 ≤ *x* ≤ 0.4) catalysts and (B) Ba_1 − *x*_Sr_*x*_Fe_0.4_Mn_0.6_O_3 − δ_ (0.6 ≤ *x* ≤ 1) catalysts**.

We investigated A-site substitution ratio (=*x*) in Ba_1 − *x*_Sr_*x*_Fe_0.4_Mn_0.6_O_3 − δ_ (*x* = 0, 0.2, 0.4, 0.6, 0.8, and 1) catalysts on the activity/selectivity for EBDH. Figure 1 and Table 4 portray the results of the activity test. As for the activity and stability of these catalysts, these perovskite catalysts deactivated gradually. The stability was increased with high Sr^2+^ content of 0.6 or 0.8 in the catalyst. Then the relation among stability, amount of carbon deposition, and the structure of catalyst was examined. First, temperature-programmed oxidation was conducted to measure the amount of carbon deposition over Ba_1 − *x*_Sr_*x*_Fe_0.4_Mn_0.6_O_3 − δ_ (0 ≤ *x* ≤ 1) catalysts after 2 h reaction. Results showed that the amount of deposited carbon was very little for each catalyst about 5 mg g-cat^−1^, and no relation was found between stability and the amount of carbon deposition as shown in Table 2. Therefore, carbon deposition does not seem to be the reason for deactivation of the catalyst. Next, the structure of as-made and used catalysts was evaluated using XRD. Figures 2A,B respectively depict XRD patterns of Ba_1 − *x*_Sr_*x*_Fe_0.4_Mn_0.6_O_3 − δ_ (0 ≤ *x* ≤ 0.4) catalysts and of Ba_1 − *x*_Sr_*x*_Fe_0.4_Mn_0.6_O_3 − δ_ (0.6 ≤ *x* ≤ 1) catalysts. From Figures 2A,B, the peak position was shifted between the fresh catalyst and used one. The shift in peak position seemed to be caused by producing oxygen vacancies. Figure 2A shows the XRD patterns of Ba_1 − *x*_Sr_*x*_Fe_0.4_Mn_0.6_O_3 − δ_ (0 ≤ *x* ≤ 0.4) catalysts include BaMnO_3_ and BaMnO_3 − δ_ after 2 h reaction. BaMnO_3 − δ_ perovskite structure had lattice vacancy in the structure. However, the structure of used Ba_1 − *x*_Sr_*x*_Fe_0.4_Mn_0.6_O_3 − δ_ (0.6 ≤ *x* ≤ 1) catalysts were almost identical structures to those of as-made catalyst derived from SrMnO_3_ structure from Figure 2B. For Figure 2A at about 2θ = 24°, small peak was observed after reaction, although the peak was not observed over the as-made catalyst. The appeared peak was attributable to the BaMnO_3 − δ_ structure. The reason for the appearance of small peak was the difference of sharing state of perovskite unit cell. The as-made catalyst has a cubic type structure with corner-sharing MnO_6_ octahedral. In contrast to the stoichiometric BaMnO_3_, oxygen-deficient BaMnO_3 − δ_ has different hexagonal/rhombohedral structures with variable ratios of corner-sharing (cubic) and face-sharing (hexagonal) layers (Adkin and Hayward, 2006, 2007). These differences produced the new peak after dehydrogenation reaction. Such created lattice vacancy under dehydrogenation atmosphere might be due to the fact that the release rate of lattice oxygen (Equation 2) was higher than the regeneration rate of lattice oxygen. Therefore, lattice oxygen in BaMnO_3_ was consumed and the BaMnO_3 − δ_ structure appeared. However, lattice vacancy was not created by low content of Ba^2+^ in the perovskite, as shown in Figure 2B. This fact might be explained by the well-balanced rates of release and regeneration of lattice oxygen. Therefore, the imbalance of release rate and regeneration rate of lattice oxygen is attributable to the deactivation of the catalyst and change of structure attributable to the reduction by EB on Ba_1 − *x*_Sr_*x*_Fe_0.4_Mn_0.6_O_3 − δ_ (0 ≤ *x* ≤ 0.4).

**Table 4:** **Selectivity to styrene, benzene and toluene for EBDH over Ba_1 − *x*_Sr_*x*_Fe_0.4_Mn_0.6_O_3 − δ_ (0 ≤ *x* ≤ 1) catalysts and Fe–K catalyst**.

**Catalyst**	**BET specific surface area/m^2^ g^−1^**	**Selectivity (0.5 h)/%**			**Selectivity (2 h)/%**		
		**Sty**	**Bz**	**Tol**	**Sty**	**Bz**	**Tol**
BaFe_0.4_Mn_0.6_ O_3 − δ_	7.1	94.3	2.0	3.7	97.0	1.1	1.9
Ba_0.8_Sr_0.2_Fe_0.4_ Mn_0.6_O_3 − δ_	6.5	95.2	2.6	2.2	96.9	0.8	2.4
Ba_0.6_Sr_0.4_Fe_0.4_ Mn_0.6_O_3 − δ_	7.9	96.3	2.1	1.6	97.6	0.5	1.9
Ba_0.4_Sr_0.6_Fe_0.4_ Mn_0.6_O_3 − δ_	9.0	96.1	2.4	1.5	98.0	0.4	1.6
Ba_0.2_Sr_0.8_Fe_0.4_ Mn_0.6_O_3 − δ_	13.1	95.4	2.9	1.7	97.8	0.6	1.7
SrFe_0.4_Mn_0.6_ O_3 − δ_	11.6	91.9	6.5	1.7	97.0	0.8	2.2
Fe–K	2.0	97.8	1.1	1.1	98.3	0.9	0.8

From these results and consideration, the Sr^2+^ substitution ratio of 0.6 was better in terms of high initial activity and stability for EBDH. However, Fe–K industrial catalyst still shows higher stability, as described above. Therefore, we controlled the stability by changing the substitution amount of Fe in Ba_0.4_Sr_0.6_Fe_*y*_Mn_1 − *y*_O_3 − δ_ (0.2 ≤ *y* ≤ 0.8) catalysts in the next section.

### Optimization of B-site substitution ratio in Ba_0.4_Sr_0.6_Fe_y_Mn_1 − y_O_3 − δ_ catalyst for EBDH

**Figure 3:**
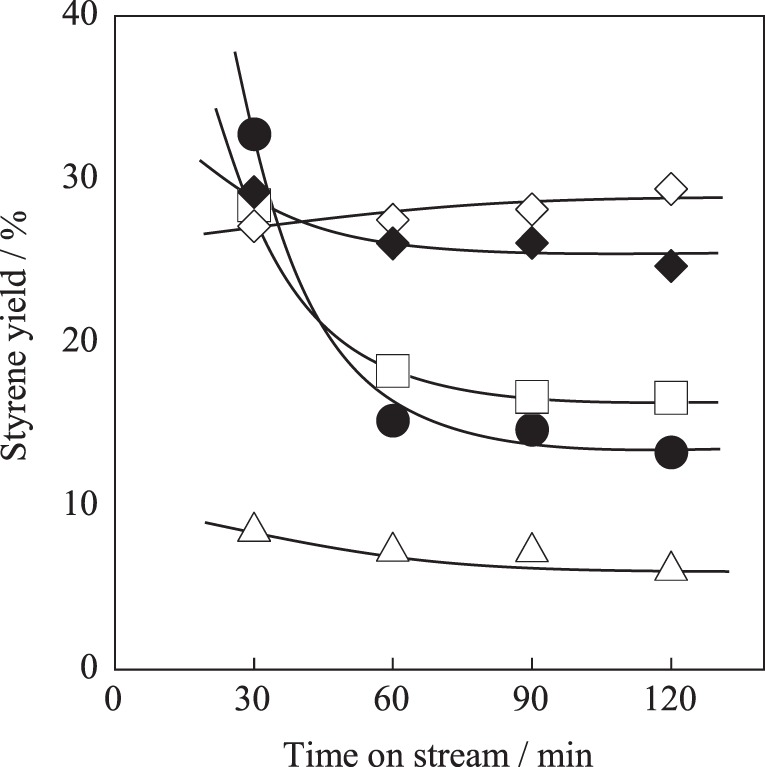
**Effect of Fe cation substitution ratio in Ba_0.4_Sr_0.6_Fe_*y*_Mn_1 − *y*_O_3 − δ_ catalyst on styrene yield: (•) *y* = 0.2, (□) *y* = 0.4, (♦) *y* = 0.6, (∆) *y* = 0.8, and (◊) Fe–K catalysts**.

**Figure 4:**
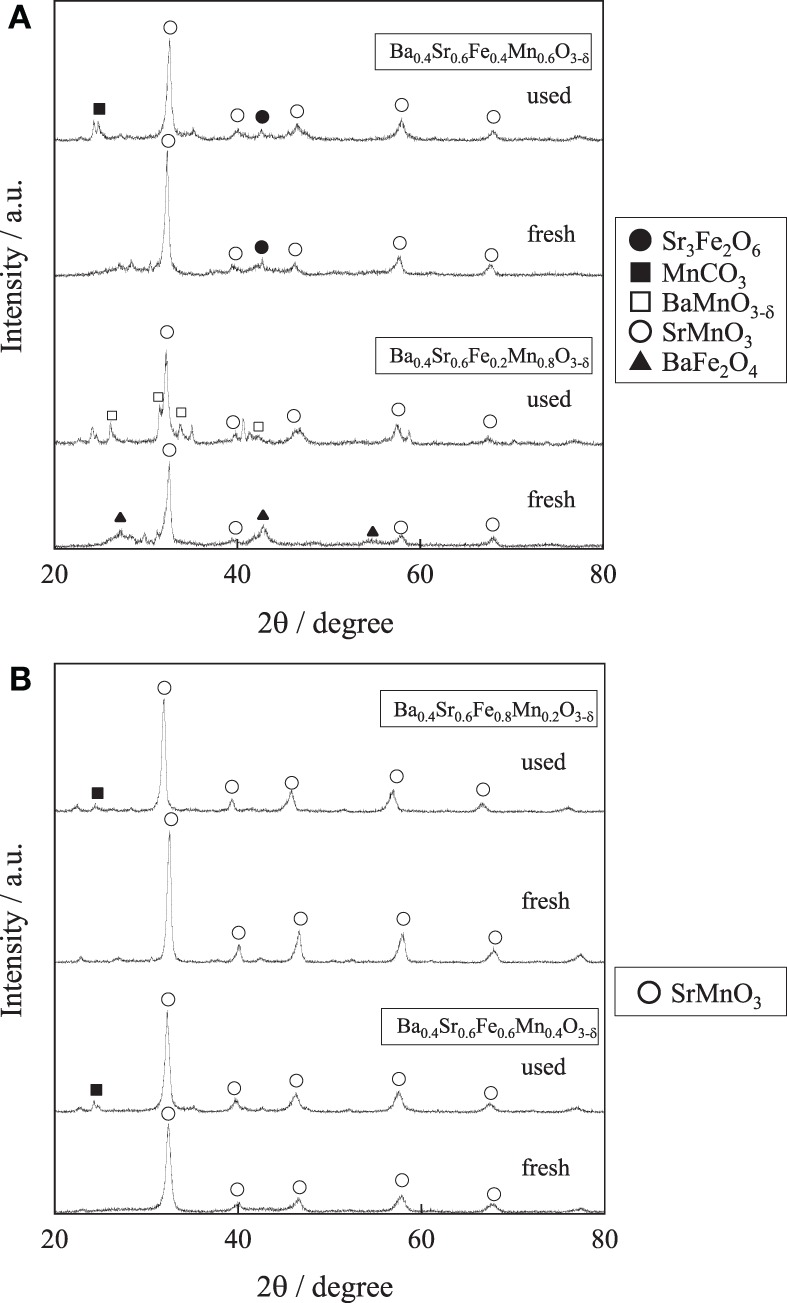
**XRD patterns for (A) Ba_0.4_Sr_0.6_Fe_*y*_Mn_1 − *y*_O_3 − δ_ (*y* = 0.2 and 0.4) catalysts and (B) Ba_0.4_Sr_0.6_Fe_*y*_Mn_1 − *y*_O_3 − δ_ (*y* = 0.6 and 0.8) catalysts**.

For further enhancement of stability, the Fe substitution ratio (*y*) in Ba_0.4_Sr_0.6_Fe_*y*_Mn_1 − *y*_O_3 − δ_ was optimized. Activity tests were performed over Ba_0.4_Sr_0.6_Fe_*y*_Mn_1 − *y*_O_3 − δ_ (*y* = 0.2, 0.4, 0.6, and 0.8) catalysts at 813 K. Figure 3 shows the styrene yield as a function of reaction time. Results showed that the stability was enhanced with increasing Fe cation substitution ratio, as we expected. Figures 4A,B show XRD patterns for these catalysts. As-made Ba_0.4_Sr_0.6_Fe_0.2_Mn_0.8_O_3 − δ_ catalyst contained perovskite-type oxide and undesired BaFe_2_O_4_ phase. After 2 h reaction, Ba_0.4_Sr_0.6_Fe_0.2_Mn_0.8_O_3 − δ_ catalyst contained many impurity phases because EB reduced the catalyst during the reaction by imbalance of release rate and the regeneration rate of lattice oxygen. However, as-made Ba_0.4_Sr_0.6_Fe_*y*_Mn_1 − *y*_O_3 − δ_ (*y* = 0.4, 0.6, and 0.8) catalysts showed characteristic peaks for perovskite-type oxide. After EBDH reaction, Ba_0.4_Sr_0.6_Fe_*y*_Mn_1 − *y*_O_3 − δ_ (*y* = 0.4, 0.6, and 0.8) catalysts showed almost identical structure to that of the as-made catalyst. Enhancement of stability was regarded as improving the regenerating rate of lattice oxygen by Fe substitution.

Table 5 presents the effect of the Fe substitution with Mn on the selectivity to products. Fe substitution increased selectivity to benzene and decreased selectivity to toluene slightly, although styrene selectivity did not change from *y* = 0.2 to *y* = 0.6 on Ba_0.4_Sr_0.6_Fe_*y*_Mn_1 − *y*_O_3 − δ_ catalysts. Miyakoshi et al. investigated the catalytic performance of partially Mn-substituted Fe–K catalysts (Miyakoshi et al., 2001). They reported that styrene selectivity was almost invariable in whole range of Mn-substitution. In this work, styrene selectivity over Ba_0.4_Sr_0.6_Fe_*y*_Mn_1 − *y*_O_3 − δ_ (0.2 ≤ *y* ≤ 0.8) catalyst was also invariable within the range: 0.2 ≤ *y* ≤ 0.6 as shown in Table 5. The trend was almost the same. The reason for low styrene selectivity over the Ba_0.4_Sr_0.6_Fe_0.8_Mn_0.2_O_3 − δ_ catalyst was significantly low styrene yield, compared with Ba_0.4_Sr_0.6_Fe_*y*_Mn_1 − *y*_O_3 − δ_ (0.2 ≤ *y* ≤ 0.6) catalysts. Low activity might be derived from low mixed conductivity of the catalyst.

**Table 5:** **Selectivity to styrene, benzene, and toluene on EBDH over Ba_0.4_Sr_0.6_Fe_*y*_Mn_1 − *y*_O_3 − δ_ (0.2 ≤ *x* ≤ 0.8) and Fe–K catalyst**.

**Catalyst**	**BET specific surface area/m^2^ g^−1^**	**Selectivity (0.5 h)/%**			**Selectivity (2 h)/%**		
		**Sty**	**Bz**	**Tol**	**Sty**	**Bz**	**Tol**
Ba_0.4_Sr_0.6_Fe_0.2_ Mn_0.8_O_3 − δ_	8.9	95.9	2.1	2.0	98.2	0.3	1.5
Ba_0.4_Sr_0.6_Fe_0.4_ Mn_0.6_O_3 − δ_	9.0	96.1	2.4	1.5	98.0	0.4	1.6
Ba_0.4_Sr_0.6_Fe_0.6_ Mn_0.4_O_3 − δ_	10.1	96.6	1.7	1.7	97.8	0.6	1.6
Ba_0.4_Sr_0.6_Fe_0.8_ Mn_0.2_O_3 − δ_	5.7	89.7	8.2	2.2	92.4	4.7	2.9
Fe–K	2.0	97.8	1.1	1.1	98.3	0.9	0.8

Among these catalysts, Ba_0.4_Sr_0.6_Fe_0.6_Mn_0.4_O_3 − δ_ catalyst showed superior catalytic performance. Comparing the activity and selectivity to styrene on Ba_0.4_Sr_0.6_Fe_0.6_Mn_0.4_O_3 − δ_ catalyst with that on Fe–K industrial catalyst, Ba_0.4_Sr_0.6_Fe_0.6_Mn_0.4_O_3 − δ_ catalyst revealed higher initial activity and almost identical selectivity, as shown in Figure 3 and Table 5.

### Characterization of surface analyses of Ba_0.4_Sr_0.6_Fe_0.6_Mn_0.4_O_3 − δ_ catalyst by X-ray photoelectron spectroscopy measurement for characterization of catalyst surface

**Figure 5:**
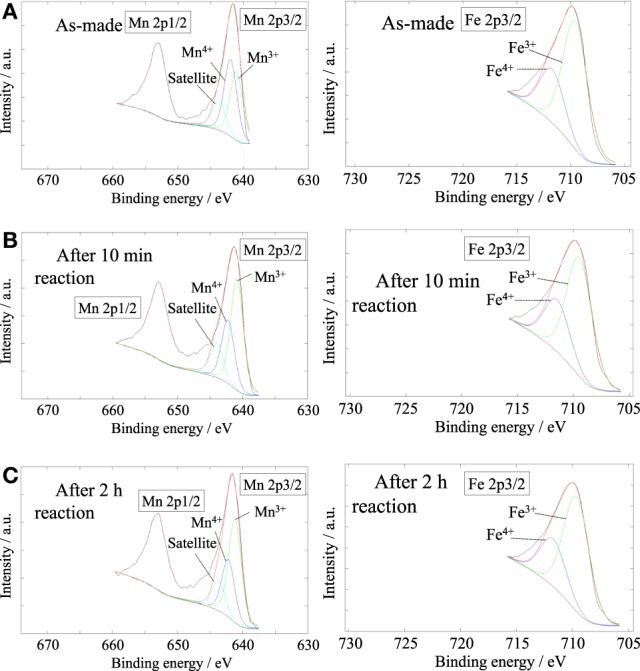
**XPS spectra of Mn2p (left) and Fe 2p3/2 (right) for Ba_0.4_Sr_0.6_Fe_0.6_Mn_0.4_O_3−δ_; **(A)** as-made, **(B)** after 10 min of reaction, **(C)** after 2 h of reaction**.

XPS measurement was carried out to evaluate the concentration of lattice vacancy in the Ba_0.4_Sr_0.6_Fe_0.6_Mn_0.4_O_3 − δ_ catalyst as-made catalyst and during EBDH reaction, and to investigate the oxidation state of B-site cation in the catalyst. XPS spectra of Mn2p and Fe2p are shown in Figure 5. Table 6 shows binding energies of Mn2p_3/2_ (Mn2p) and Fe2p_3/2_ (Fe2p) core-levels of as-made catalyst, the catalyst after 10 min reaction and the catalyst after 2 h reaction. The concentration of oxygen vacancy at the surface calculated from the surface atomic ratio is shown in Table 6. Here, the peak percentage of components is in parenthesis. As for as-made catalyst, the peak of Mn2p included two components at 641.94 and 640.98 eV. Former and latter peaks were attributed to Mn^4+^ and Mn^3+^, respectively (Carver et al., 1972; Oku and Hirokawa, 1976; Ponce et al., 2000). The peak of Fe2p included components at 711.71 and 709.56 eV which were attributed to Fe^4+^ and Fe^3+^, respectively (Ghaffari et al., 2012). The peak percentage of components was as follows; Mn^4+^: 51.5%, Mn^3+^: 48.5%, Fe^4+^: 30.3%, and Fe^3+^: 69.7%. The concentration of oxygen vacancy; 3–δ was 2.88. The composition of perovskite which has no lattice vacancy, is ABO_3_, in other words d is 0. In this work, the low valence cation; Ba was doped in A-site of perovskite-type oxides, in order to maintain the electrical neutrality, the electrical charge unbalance could be compensated via following ways: (a) the increase of valence state of B-site metal cation and (b) the production of lattice vacancy in the structure. Therefore, the result indicated the presence of lattice vacancy.

**Table 6:** **Binding energy and surface lattice vacancy of Ba_0.4_Sr_0.6_Fe_0.6_Mn_0.4_O_3 − δ_ catalyst under various reaction conditions**.

**Condition**	**Mn2p/eV**		**Fe2p/eV**		**Surface composition**
	**Mn^4^+**	**Mn^3^+**	**Fe^4^+**	**Fe^3^+**	
As-made	641.94 (51.5)	640.98 (48.5)	711.71 (30.3)	709.56 (69.7)	Ba_0.57_Sr_0.48_Fe_0.61_Mn_0.50_O_2.88_
After 10 min reaction	642.25 (36.4)	640.86 (63.6)	711.59 (29.5)	709.49 (70.5)	Ba_0.69_Sr_0.47_Fe_0.54_Mn_0.47_O_2.83_
After 2 h reaction	642.19 (35.9)	641.17 (64.1)	711.71 (30.3)	709.68 (69.7)	Ba_0.81_Sr_0.20_Fe_0.63_Mn_0.51_O_2.87_

Peak percentage of components is in parenthesis.

As for the Ba_0.4_Sr_0.6_Fe_0.6_Mn_0.4_O_3 − δ_ catalyst after 10 min reaction, the peak of Mn2p was 642.25 and 640.86 eV. These values were attributed to the Mn^4+^, Mn^3+^, respectively in Figure 5B. The Fe2p peak can be divided into two peaks, namely Fe^4+^ at 711.59 eV and Fe^3+^ at 709.49 eV. The peak percentage of components was as follows; Mn^4+^: 36.4%, Mn^3+^: 63.6%, Fe^4+^: 29.5%, and Fe^3+^: 70.5%. The Ba_0.4_Sr_0.6_Fe_0.6_Mn_0.4_O_3 − δ_ catalyst was reduced by EB and high valence cation of Mn^4+^ was reduced to lower valence cation of Mn^3+^. While, the oxidation state of Fe did not almost change after the reaction. In fact, the Fe cation in the Ba_0.4_Sr_0.6_Fe_0.6_Mn_0.4_O_3 − δ_ catalyst was not reduced by EB. The concentration of oxygen vacancy; _3 − δ_ was 2.83. The catalyst also had lattice vacancy in the perovskite structure. While, the peak of Mn2p and Fe2p in the Ba_0.4_Sr_0.6_Fe_0.6_Mn_0.4_O_3 − δ_ catalyst after 2 h reaction contained Mn^4+^, Mn^3+^, Fe^4+^, and Fe^3+^. The mixed valence of Mn and Fe was not almost changed, compared with the catalyst after 10 min reaction. The peak percentage of components was as follows; Mn^4+^: 35.9%, Mn^3+^: 64.1%, Fe^4+^: 30.3%, and Fe^3+^: 69.7%.

We considered the role of Fe from the results of XPS results. Preliminarily studies showed that redox-type mechanism proceeded on the catalyst; (Equation 2) oxidative dehydrogenation of ethylbenzene using lattice oxygen and (Equation 3) regenerating lattice vacancy by H_2_O (Watanabe et al., 2011, 2013). In this work, Mn-based catalyst showed high initial activity, but low stability. While, stability of the catalyst was improved by Fe substitution of Ba_0.4_Sr_0.6_Fe_*y*_Mn_1 − *y*_O_3 − δ_ catalyst. Therefore, we proposed that the role of Mn and Fe might be the site for releasing lattice oxygen and the site for regenerating lattice vacancy, respectively. The oxidation state of Fe and Mn on Ba_0.4_Sr_0.6_Fe_0.6_Mn_0.4_O_3 − δ_ catalyst was Mn^4+^/Mn^3+^ and Fe^4+^/Fe^3+^, respectively. So, the aromatic ring of EB was adsorbed onto Mn^4+^. Then, styrene and H_2_O were produced via oxidative dehydrogenation of ethylbenzene, following that Mn^4+^ and Fe^4+^ were reduced to Mn^3+^ and Fe^3+^. Co-feeding steam was adsorbed on reduced Fe^3+^ site. Lattice oxygen was regenerated by oxidation of Fe^3+^ to Fe^4+^ and of Mn^3+^ to Mn^4+^. Summarily, the role of Fe was considered to be the promotion of regenerating lattice oxygen derived from high redox property of Fe^4+^/Fe^3+^.

### Dehydrogenation activity and robustness of Ba_0.4_Sr_0.6_Fe_0.6_Mn_0.4_O_3 − δ_ catalyst under low-temperature or low steam/EB condition

**Figure 6:**
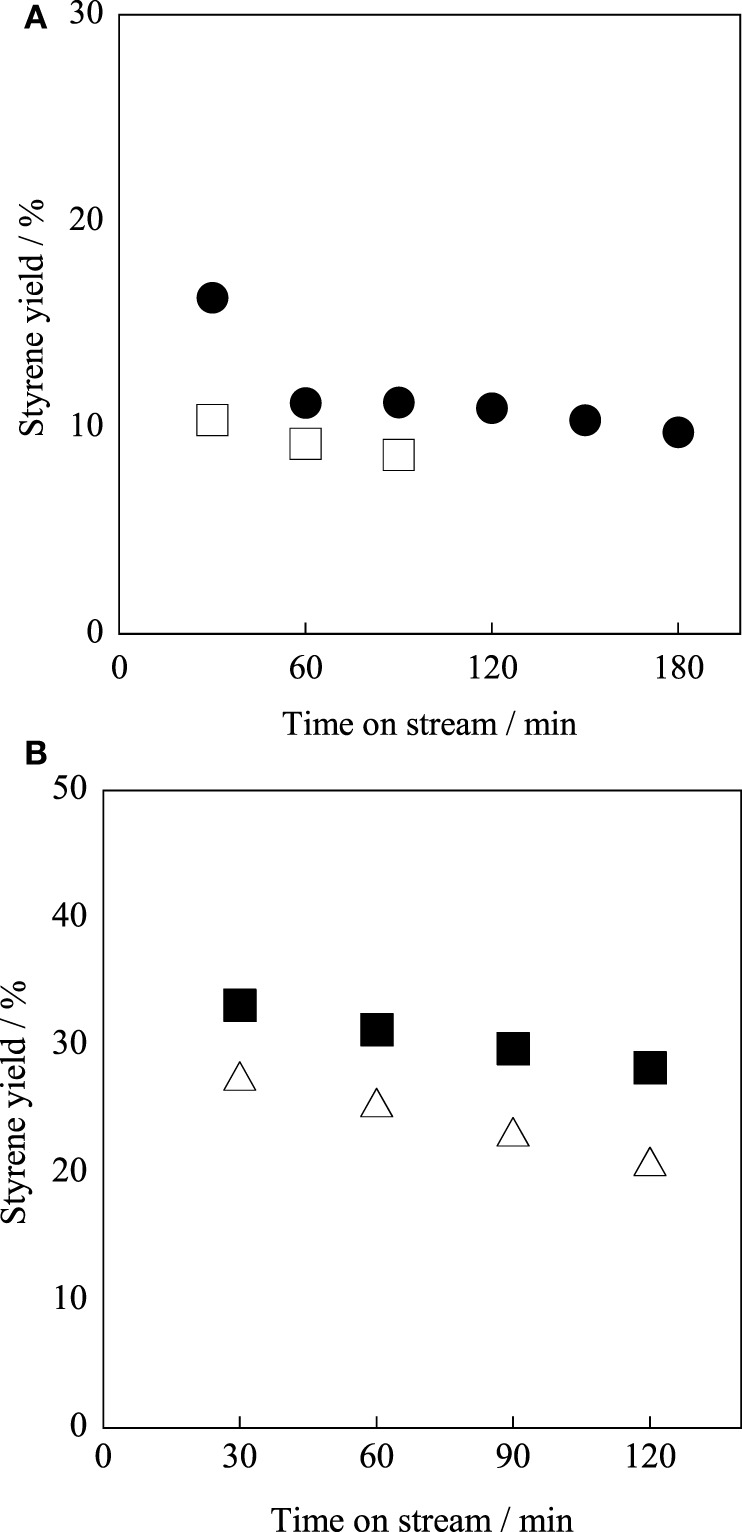
**Catalytic activity tests over (filled symbols) Ba_0.4_Sr_0.6_Fe_0.6_Mn_0.4_O_3 − δ_ and (open symbols) Fe–K catalysts (A) at 783 K and under steam/EB molar ratio of 12, (B) at 813 K under steam/EB molar ratio of 2**.

To examine the dehydrogenation activity and robustness of the Ba_0.4_Sr_0.6_Fe_0.6_Mn_0.4_O_3 − δ_ catalyst under low temperature or low steam/EB condition, catalytic activity tests were conducted. The Fe–K oxide catalyst was used as the control. Figure 6A presents results for activity tests at 783 K (i.e., lower temperature) under steam/EB of 12. The styrene yield of the Ba_0.4_Sr_0.6_Fe_0.6_Mn_0.4_O_3 − δ_ catalyst was 16.3% and that of the Fe–K catalyst was 10.4% at 30 min. The initial activity of the Ba_0.4_Sr_0.6_Fe_0.6_Mn_0.4_O_3 − δ_ catalyst was 1.57 times higher than that of the Fe–K oxide catalyst at such a low temperature.

Next, the robustness of the Ba_0.4_Sr_0.6_Fe_0.6_Mn_0.4_O_3 − δ_ catalyst for low steam/EB operation was examined, and catalytic activity tests were performed at 813 K with steam/EB molar ratio of 2. Figure 6B presents results for the activity test over the Ba_0.4_Sr_0.6_Fe_0.6_Mn_0.4_O_3 − δ_ and the Fe–K catalysts under the condition of steam/EB molar ratio of 2. From Figure 6B, the Ba_0.4_Sr_0.6_Fe_0.6_Mn_0.4_O_3 − δ_ catalyst showed high robustness under low steam conditions. However, the activity of Fe–K oxide catalyst gradually decreased with time on stream. These results might be derived from the superior ability for steam activation over the Ba_0.4_Sr_0.6_Fe_0.6_Mn_0.4_O_3 − δ_ catalyst compared to that of the Fe–K catalyst. For the Fe–K catalyst, low steam/EB conditions caused a low regeneration rate of lattice oxygen, and deactivation was observed.

**Figure 7:**
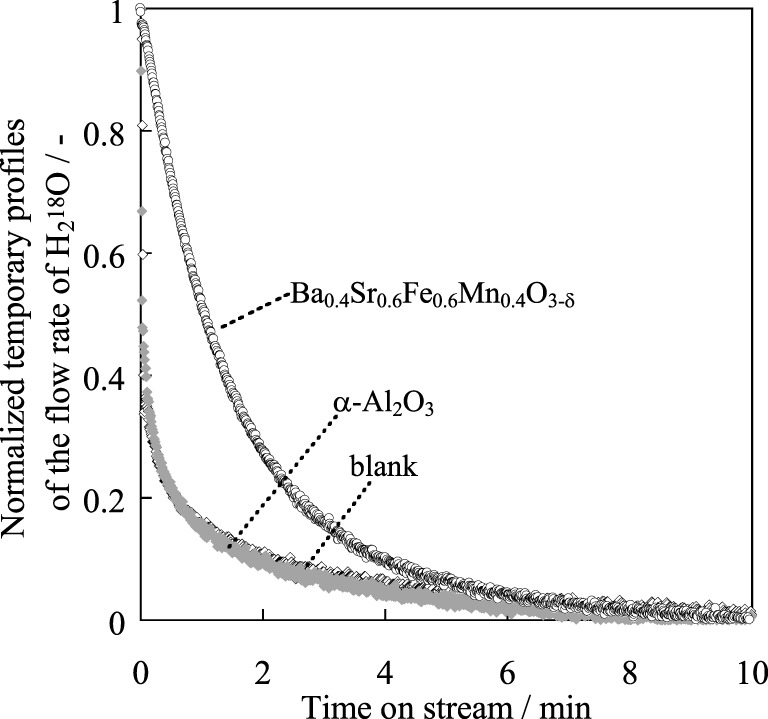
**Normalized temporary profile of H^18^_2_O flow rate over α-Al_2_O_3_ or Ba_0.4_Sr_0.6_Fe_0.6_Mn_0.4_O_3 − δ_ catalyst**.

The reason for the higher activity of the Ba_0.4_Sr_0.6_Fe_0.6_Mn_0.4_O_3 − δ_ was investigated in terms of the redox property of the catalyst. An isotope exchange reaction from ^16^O_lat_ to ^18^O_lat_ in the catalyst by switching H^16^_2_O flow to H^18^_2_O at 783 K was conducted over the Ba_0.4_Sr_0.6_Fe_0.6_Mn_0.4_O_3 − δ_ catalyst. We have already found that Fe–K catalyst works on a redox mechanism during EBDH (Sekine et al., 2008). Figure 7 presents temporary profiles of the flow rate of H^18^_2_O by the time that the stable flow rate of H^18^_2_O was confirmed. Here, time “0” was the starting time at which H^18^_2_O (30%) and H^16^_2_O (70%) were supplied to the catalyst. H^18^_2_O was monitored sooner after the feeding of H^18^_2_O on a blank test and over α-Al_2_O_3_ as a non-redox control, respectively. The isotope exchange reaction did not proceed over α-Al_2_O_3_, in accordance with the profile of the blank test. Different from α-Al_2_O_3_, a slow exchange profile was observed over Ba_0.4_Sr_0.6_Fe_0.6_Mn_0.4_O_3 − δ_ catalyst and the isotope exchange continued for about 10 min from the switching gas. This phenomenon shows that an exchange reaction (Equation 7) proceeds only on Ba_0.4_Sr_0.6_Fe_0.6_Mn_0.4_O_3 − δ_ catalyst.

(7) $${\text{H}}_{2}^{18}\text{O}{+}^{16}{\text{O}}_{\text{lat}}^{2-}↔{\text{H}}_{2}^{16}\text{O}{+}^{18}{\text{O}}_{\text{lat}}^{2-}$$

Here, ^16^O^2−^_lat_ and ^18^O^2−^_lat_ denotes lattice oxygen with a mass number of 16 and 18. Then amounts of exchanged lattice oxygen were calculated. The amount was large compared with the amount of surface lattice oxygen. Bulk lattice oxygen was exchanged according to the following equation (Equations 8 and 9).

(8) $${\text{H}}_{2}^{18}\text{O}{+}^{16}{\text{O}}_{\text{lat},\text{ surf}}^{2-}↔{\text{H}}_{2}^{16}\text{O}{+}^{18}O_{\text{lat},\text{ surf}}^{2-}$$

(9) $${}^{18}{\text{O}}_{\text{lat},\text{ surf}}^{2-}+{\text{V}}_{\text{ox},\text{ bulk}}{↔}^{16}{\text{O}}_{\text{lat},\text{ bulk}}^{2-}+{\text{V}}_{\text{ox},\text{ surf}}$$

**Figure 8:**
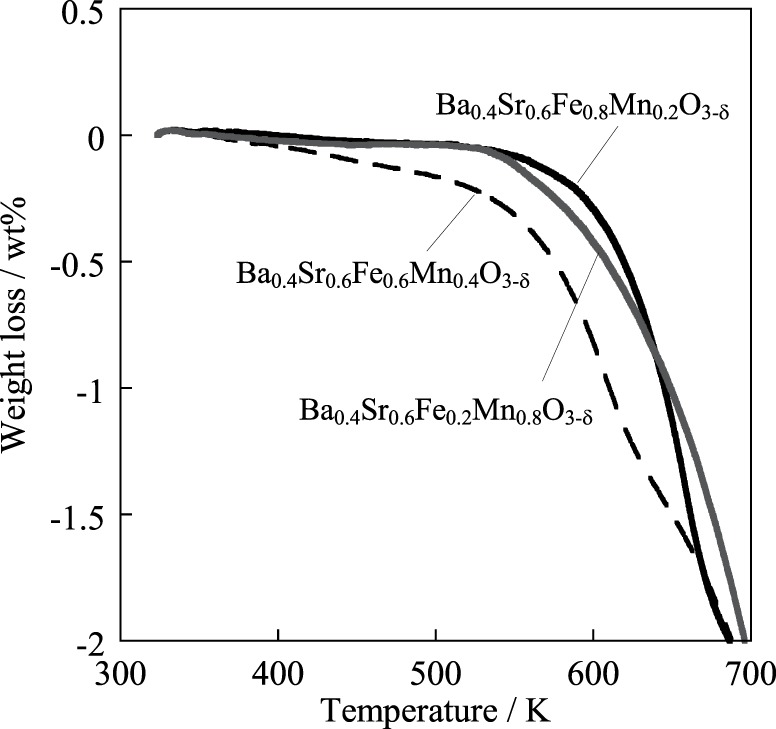
**Reduction behavior of Ba_0.4_Sr_0.6_Fe_*y*_Mn_1 − *y*_O_3 − δ_ (*y* = 0.2, 0.6, 0.8) catalysts under 10 vol% H_2_ atmosphere**.

Here, “surf” and “bulk” respectively signify the surface and bulk of the catalyst. The surface exchange reaction proceeded on the catalyst, and then the surface lattice oxygen diffused to bulk with redox of the catalyst. Figure 8 shows the result of H_2_-temperature programmed reduction (TPR) measurement of Ba_0.4_Sr_0.6_Fe_*y*_Mn_1 − *y*_O_3 − δ_ (*y* = 0.2, 0.6, 0.8) catalysts. The weight loss of the catalyst was measured by heating to 1173 K (10 K min^−1^) under 10 vol% H_2_ atmosphere. The start of the reduction was considered to reflect the consumption of lattice oxygen on the vicinity of the surface. From this Figure, Ba_0.4_Sr_0.6_Fe_0.8_Mn_0.2_O_3 − δ_ catalyst was less likely to be reduced. Namely, reducibility of the catalyst was low, comparing with other catalysts. Low releasing ability of lattice oxygen might cause low activity for dehydrogenation of ethylbenzene. Based on these results, the Ba_0.4_Sr_0.6_Fe_0.6_Mn_0.4_O_3 − δ_ catalyst enabled redox at low temperature of 783 K. Consequently, high activity is apparently derived from high redox property of the Ba_0.4_Sr_0.6_Fe_0.6_Mn_0.4_O_3 − δ_ catalyst.

**Figure 9:**
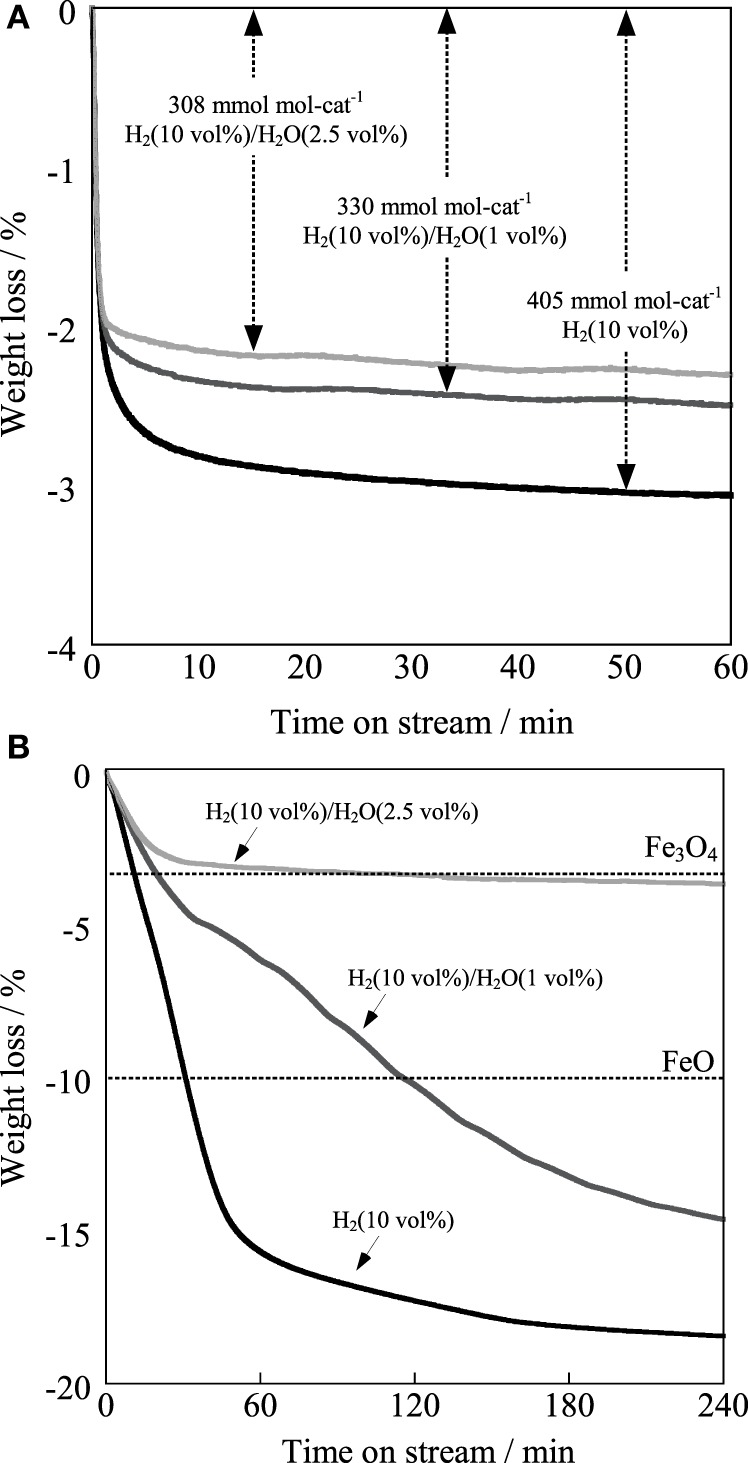
**Reduction profiles of (A) Ba_0.4_Sr_0.6_Fe_0.6_Mn_0.4_O_3 − δ_ catalyst and (B) Fe–K catalyst at 783 K under H_2_ (10 vol%) atmosphere in the absence of H_2_O, H_2_/H_2_O (10 vol%/1 vol%) atmosphere and H_2_/H_2_O (10 vol%/2.5 vol%) atmosphere**.

For elucidating the greater robustness of the Ba_0.4_Sr_0.6_Fe_0.6_Mn_0.4_O_3 − δ_ catalyst than that of the Fe–K catalyst, redox properties of the Ba_0.4_Sr_0.6_Fe_0.6_Mn_0.4_O_3 − δ_ and the Fe–K catalysts were examined using thermogravimetric measurement under H_2_ atmosphere and H_2_/H_2_O atmosphere. Figure 9A portrays the thermogravimetric profile of the Ba_0.4_Sr_0.6_Fe_0.6_Mn_0.4_O_3 − δ_ catalyst under various partial pressures at 783 K and Figure 9B shows that of the Fe–K catalyst under the same condition. From Figure 9A, 3.1 wt% of the Ba_0.4_Sr_0.6_Fe_0.6_Mn_0.4_O_3 − δ_ catalyst was reduced under 10 vol% H_2_/N_2_ atmosphere. The reduced amount was equivalent to 405 mmol mol-cat^−1^, which was 13.5% of lattice oxygen in perovskite-type oxide. However, 2.5 wt% of the Ba_0.4_Sr_0.6_Fe_0.6_Mn_0.4_O_3 − δ_ was reduced under 10 vol% H_2_ and 1 vol% H_2_O. The value corresponded to 330 mmol mol-cat^−1^ of lattice oxygen, which was 11% of lattice oxygen in perovskite-type oxide. Further increase of the partial pressure of steam decreased the value to 308 mmol mol-cat^−1^ under 10 vol% H_2_ and 2.5 vol% H_2_O. The lattice oxygen of 10.3% was released under this condition. Figure 9B showed that the Fe–K was reduced to Fe metal under 10 vol% H_2_/N_2_ atmosphere. Also, the Fe–K was reduced to Fe metal under 10 vol% H_2_ and 1.0 vol% H_2_O. Compared with the case in the absence of H_2_O (10 vol% H_2_/N_2_), the reduction rate was suppressed by H_2_O. Even if the partial pressure of steam further increased, the Fe–K was reduced to Fe_3_O_4_ and additional reduction gradually proceeded from Fe_3_O_4_ to Fe metal with reaction time under 10 vol% H_2_ and 2.5 vol% H_2_O condition. The nature of the structural stability of the Fe–K catalyst was low in reducing atmosphere. From these results, coexisting H_2_O in the atmosphere affected the reduction behavior of the Ba_0.4_Sr_0.6_Fe_0.6_Mn_0.4_O_3 − δ_: i.e., reduction was suppressed by H_2_O compared to the case in the absence of H_2_O. We confirmed that the regeneration of consumed lattice oxygen by H_2_O proceeded on the catalyst in our previous study (Watanabe et al., 2011). Therefore, the suppression of the reduction was derived from the increased regeneration rate of the consumed lattice oxygen in perovskite oxide by H_2_O. These results showed that the Ba_0.4_Sr_0.6_Fe_0.6_Mn_0.4_O_3 − δ_ catalyst revealed high robustness under a reductive atmosphere and activated H_2_O more easily than the Fe–K catalyst.

For low reaction temperature and low steam/EB reaction conditions, the Ba_0.4_Sr_0.6_Fe_0.6_Mn_0.4_O_3 − δ_ catalyst had high activity and robustness. Results show that the Ba_0.4_Sr_0.6_Fe_0.6_Mn_0.4_O_3 − δ_ is a promising catalyst for industrial dehydrogenation processes.

## Conclusion

For EBDH, the substitution of Ba^2+^ for Sr^2+^over Ba_1 − *x*_Sr_*x*_Fe_*y*_Mn_1 − *y*_O_3 − δ_ (*x* = 0, 0.2, 0.4, 0.6, 0.8, and 1, *y* = 0, 0.2, 0.4, 0.6, and 0.8) catalysts enhanced the initial activity for EBDH, but activity decreased with time on stream. Optimization of B-site substitution ratio of Fe in Ba_0.4_Sr_0.6_Fe_*y*_Mn_1 − *y*_O_3 − δ_ catalyst was conducted, and results showed that the stability for EBDH was improved by an increase of Fe substitution ratio. Optimized Ba_0.4_Sr_0.6_Fe_0.6_Mn_0.4_O_3 − δ_ catalyst showed high styrene yield of 29.2 % and selectivity to styrene of 96.6% at 813 K. The dehydrogenation activity and robustness of Ba_0.4_Sr_0.6_Fe_0.6_Mn_0.4_O_3 − δ_ catalyst were investigated under low-temperature and low steam/EB reaction conditions. Consequently, the catalyst of Ba_0.4_Sr_0.6_Fe_0.6_Mn_0.4_O_3 − δ_ revealed high activity and superior robustness under these severe conditions derived from high redox property of the catalyst. From the evaluation of redox property of the Ba_0.4_Sr_0.6_Fe_0.6_Mn_0.4_O_3 − δ_ catalyst, thermogravimetric profile was measured under H_2_/H_2_O atmosphere at 783 K. The result showed that reduction was suppressed by the presence of H_2_O, implied that the lattice oxygen in perovskite-structure was consumed by H_2_, subsequently the consumed lattice oxygen was regenerated by H_2_O. Additional support for this result was confirmed by H_2_O surface exchange reaction. The lattice oxygen was exchanged with steam by redox of the catalyst at low temperature of 783 K.

Therefore, Ba_0.4_Sr_0.6_Fe_0.6_Mn_0.4_O_3 − δ_ was identified as a promising catalyst for EBDH with steam under severe conditions.

### Conflict of interest statement

The authors declare that the research was conducted in the absence of any commercial or financial relationships that could be construed as a potential conflict of interest.
